# Toll-Like Receptor Signaling and Immune Regulatory Lymphocytes in Periodontal Disease

**DOI:** 10.3390/ijms21093329

**Published:** 2020-05-08

**Authors:** Yingzhi Gu, Xiaozhe Han

**Affiliations:** 1 Department of Immunology and Infectious Diseases, The Forsyth Institute, 245 First Street, Cambridge, MA 02142, USA; ygu@forsyth.org; 2 Department of Oral Medicine, Infection and Immunity, Harvard School of Dental Medicine, Boston, MA 02115, USA

**Keywords:** periodontal disease, RANKL, Breg, Treg, TLR

## Abstract

Periodontitis is known to be initiated by periodontal microbiota derived from biofilm formation. The microbial dysbiotic changes in the biofilm trigger the host immune and inflammatory responses that can be both beneficial for the protection of the host from infection, and detrimental to the host, causing tissue destruction. During this process, recognition of Pathogen-Associated Molecular Patterns (PAMPs) by the host Pattern Recognition Receptors (PRRs) such as Toll-like receptors (TLRs) play an essential role in the host–microbe interaction and the subsequent innate as well as adaptive responses. If persistent, the adverse interaction triggered by the host immune response to the microorganisms associated with periodontal biofilms is a direct cause of periodontal inflammation and bone loss. A large number of T and B lymphocytes are infiltrated in the diseased gingival tissues, which can secrete inflammatory mediators and activate the osteolytic pathways, promoting periodontal inflammation and bone resorption. On the other hand, there is evidence showing that immune regulatory T and B cells are present in the diseased tissue and can be induced for the enhancement of their anti-inflammatory effects. Changes and distribution of the T/B lymphocytes phenotype seem to be a key determinant of the periodontal disease outcome, as the functional activities of these cells not only shape up the overall immune response pattern, but may directly regulate the osteoimmunological balance. Therefore, interventional strategies targeting TLR signaling and immune regulatory T/B cells may be a promising approach to rebalance the immune response and alleviate bone loss in periodontal disease. In this review, we will examine the etiological role of TLR signaling and immune cell osteoclastogenic activity in the pathogenesis of periodontitis. More importantly, the protective effects of immune regulatory lymphocytes, particularly the activation and functional role of IL-10 expressing regulatory B cells, will be discussed.

## 1. Introduction

Periodontitis is one of the most predominant oral diseases that affect the majority of the population worldwide. Consistent with current knowledge of pathophysiology, in periodontal disease, three forms of periodontitis can be identified: necrotizing periodontitis [1], periodontitis as a manifestation of systemic disease [2], and what was previously considered separately to be “chronic” or “aggressive” form of the disease is now classified together as a new classification of “periodontitis” [3,4,5,6,7]. Periodontitis is known to be triggered by periodontal bacteria colonized at the host–microbe interface [8]. Various studies have identified keystone microbial species that are strongly associated with the onset and progress of periodontitis, such as *Porphyromonas gingivalis (P. gingivalis*) [9], *Aggregatibacter actinomycetemcomitans* (*A. actinomycetemcomitans)* [10], *Tannerella forsythia (T. forsythia)* [11], *Fusobacterium nucleatum (F. nucleatum)* [12], *Treponema denticola (T. denticola)* [13], and *Actinomyces viscosus (A. viscosus)* [14].

**Figure 1:**
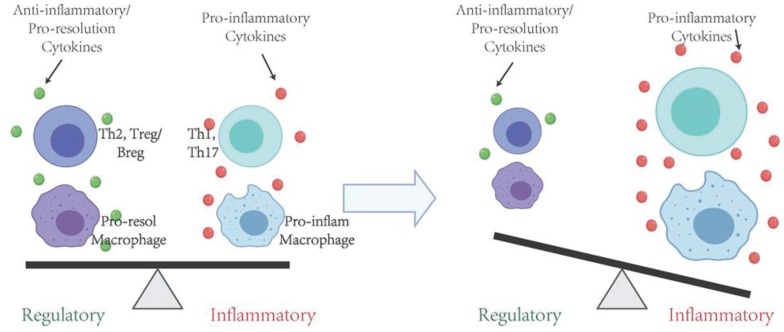
Immune responses directly contribute to the pathogenesis of periodontitis. A balanced pro- and anti-inflammatory responses need to be achieved to maintain tissue homeostasis. If the pro-inflammatory subtype of cells is predominantly persisted, it is inclined towards tissue destruction and bone resorption. Conversely, if the anti-inflammatory and pro-resolving lineages are predominantly developed in a timely fashion, inflammation will be controlled, and tissues will be repaired or regenerated.

Although certain bacteria are considered "pathogens" due to their strong association with periodontal disease, they are also found in healthy sites of diseased patients or periodontal sites of healthy individuals. Therefore, none of these bacteria can be singled out as the cause of the periodontal disease because they have to adapt into the biofilm to form an organized microbial community, evolving towards a dysbiotic microbiota, eventually causing heightened periodontal inflammation and tissue destruction. While specific components or byproducts of bacteria, such as extracellular vesicles [15,16], enzymes (collagenase, protease and hyaluronidase) [17,18,19], toxins (such as leukotoxin) [20] and their metabolites (such as hydrogen sulfide) [21] may moderately disrupt periodontal tissue, the damage elicited by the adverse interaction between the subgingival biofilm and the host inflammatory immune response is considered the main cause of periodontal pathogenesis, with more substantial and persistent soft and hard tissue destruction [22,23]. There is now strong evidence that periodontitis is an inflammatory disease triggered by the host immune response to the microorganisms associated with periodontal biofilms, or their byproducts such as lipopolysaccharide (LPS), lipoprotein acids [24,25,26,27,28]. Such imbalance of pro-inflammatory and anti-inflammatory host cellular responses are considered a key element in disease pathogenesis and tissue damage (Figure 1). 

There is a sequential event of the innate and adaptive immune responses leading to pathological alveolar bone resorption. After the acute inflammation is established, the recruitment of innate and adaptive immune cells and infiltration into the periodontal tissues mark a transition to the resolution phase or chronic inflammation. Affected by a series of environmental factors and the interactions of cellular and molecular components inherent to the host, different effector cell lineages may dominate the presence in the tissue, which determines the clinical outcome of the disease. If the pro-inflammatory subtype of cells is predominantly persisted, it is inclined towards tissue destruction and bone resorption. Conversely, if the anti-inflammatory and pro-regeneration lineages are predominantly developed in a timely fashion, inflammation will be resolved, and tissues will be repaired or regenerated.

## 2. Toll-Like Receptor (TLR) Signaling in the Etiology of Periodontitis

Ample studies have demonstrated that the initial host immune and inflammatory responses in periodontal disease were orchestrated by epithelial keratinocytes and fibroblasts of the periodontal connective tissue. Epithelial cells and gingival fibroblasts interact directly with microorganisms or their byproducts, generate and secrete molecular signals to trigger inflammation and attract immune cells [29,30]. Host cells recognize microorganisms through the interaction of Pattern Recognition Receptors (PRRs) that are constitutively expressed in the cell membrane of host cells, with the pathogen-associated molecular patterns (PAMP) presented by the microorganisms. These PAMPs are cell surface molecules that are preferentially related to pathogens and are not present in host cells, including lipopolysaccharide (LPS) (the main component of Gram-negative cell walls) and lipoteichoic acid (LTA) (the main component of cell walls of Gram-positive bacteria) [31]. The prototype membrane-bound PAMP receptor is in the Toll-like receptor (TLR) family, a type of PRRs that is considered to be the critical bridge linking innate and adaptive immunity [32]. Many studies have revealed that TLRs can recognize periodontal pathogens and regulate the host innate and adaptive immune response in periodontal disease [33,34,35]. To date, ten TLRs (TLR1 to TLR10) have been identified in humans. Each TLR is located in a specific compartment of the cell and can sense different PAMPs. T and B cells have been shown to express a number of TLRs (such as TLR 1, 2, 4, and 9) and respond to TLR ligands [36,37]. LPS is a potent ligand for TLR4 [38,39]. Lipoproteins produced by periodontal bacteria and *P. gingivalis* LPS can be recognized as ligands for TLR2 [40,41,42]. One study showed that *P. gingivalis* can activate both of the TLR2 and TLR4 pathways, leading to excessive production of pro-inflammatory cytokines and chemokines in monocytes [43]. TLR9 is known to be activated by microbial nucleic acids unmethylated CpG Oligodeoxynucleotide DNA (CpG) that can lead to periodontal inflammation and bone loss [44]. Stimulation by different types of bacteria in periodontal biofilm can induce the expression of different TLRs. The binding of pathogen-derived ligands to membrane-bound TLRs lead to the dimerization of TLRs and the subsequent activation of the downstream signaling pathways associated with inflammation and osteoclastogenesis.

The dimerization of TLR triggers the recruitment of various protein kinases in the cytoplasmic end of the receptor, and the classical inflammatory pathway is activated, leading to the upregulation of pro-inflammatory transcription factors (such as NFκB and AP-1) [45,46]. Various pro-inflammatory molecules, such as prostaglandin E-2 and leukotriene A-4 are produced. At the same time, TLR recognition of microbial ligands leads to the secretion of important molecular mediators of inflammation, including inflammatory cytokines (such as TNFα and IL-1β) and chemokines (such as CXCL8/IL-8, CCL2, CCL3 and CCL5), which promote the migration and infiltration of inflammatory cells into diseased periodontal tissues, such as dendritic cells, macrophages, and T and B cells [47,48]. Recently, a review of clinical human in vitro and in vivo studies reported a correlation between IFNγ in the crevicular fluid and periodontitis, potentially mediating the modulation of osteoclastogenesis [49]. During these processes, the infiltration of a large number of inflammatory cells is achieved by the degradation of the extravascular matrix around the blood vessels by enzymes such as matrix metalloproteinase (MMP) [50,51]. Under the influence of a large number of inflammatory signals, gingival fibroblasts and periodontal ligament fibroblasts destroy the fibrous components of the extracellular matrix of periodontal tissue by increasing the local production and activity of MMPs [52], leading to the destruction of periodontal soft tissues. 

On the other hand, the role of TLR signaling on osteoclastogenesis and periodontal bone loss has also been extensively investigated. Ohgi et al. stimulated bone marrow macrophages (BMMs) with synthetic ligands for TLR2 (Pam3CSK4) or TLR4 (Lipid A), with or without receptor activator of nuclear factor kappa-B ligand (RANKL), and assessed for osteoclastogenesis by tartrate-resistant acid phosphatase (TRAP) staining, and found that osteoclastogenesis is promoted under the coexistence of oxidized low-density lipoprotein by TLR2-induced upregulation of Lectin-like oxidized low-density lipoprotein receptor 1 in mouse bone marrow cells [53]. Kishimoto et al. injected *Escherichia coli* peptidoglycan (PGN) or *Staphylococcus aureus* PGN with or without LPS into mouse gingiva, and histologically assessed alveolar bone resorption by TRAP staining. The results showed that Gram-positive or Gram-negative PGN worked synergistically with LPS to induce bone resorption and osteoclastogenesis, possibly by coordinating the effects of TLR2 and TLR4 signaling [54]. Zhang et al. investigated the direct effects of the periodontal pathogen *P. gingivalis* on osteoclast differentiation and showed that *P. gingivalis* differentially modulates RANKL-induced osteoclast formation through the modulation of TLR2/myeloid differentiation factor 88 (MyD88) [55]. Moreover, Yu et al. revealed significantly decreased bone loss and TRAP-positive cells in TLR2 KO mice as compared to WT mice in ligature-induced peri-implantitis and periodontitis, suggesting that TLR2 mediates bone loss in both peri-implantitis and periodontitis [56]. These findings strongly suggested the key roles of TLR signaling in the induction of periodontal inflammation and bone resorption.

## 3. Immune T and B Cells in Periodontitis Pathogenesis 

Within the connective tissue of the periodontal infection site, there is a dense mononuclear inflammatory infiltrate containing all the cellular components of the immune network. In recent years, increasing evidence has substantiated that the inflammatory activation of the immune system in periodontitis cause heightened pathological osteoclastogenesis and alveolar bone destruction. Such close connection and interaction between the immune system and bone metabolism has been referred to as "osteoimmunology" [57,58,59]. Many studies have shown that the host immune response plays a key role in stimulating osteoclast differentiation and promoting bone resorption in periodontal disease [60,61,62,63,64].

It is now clear that the host response to bacteria involving activated T and B lymphocytes and these cells contribute to the pathogenesis of periodontitis bone resorption. Previous studies have shown that a large number of T and B lymphocytes were infiltrated in gingival tissue in an antigen-specific manner [65,66,67]. Specifically, key links between T cells and bone resorption have been established [68]. Yoshie and colleagues [69] implicated the pathogenesis of activated T lymphocytes and periodontal disease. Similar results have been observed in experimental mouse models, suggesting that T cells and their response to oral infections by *P. gingivalis* help advance bone remodeling in the direction of net bone loss [70]. Kawai and colleagues used an experimental adoptive T cell transfer periodontal disease model to show that Th1 cells combined with B7 co-stimulation appeared to trigger inflammatory bone resorption [71]. This bone resorption can be eliminated by the use of a fusion protein (CTLA4Ig), which interferes with the co-stimulatory interaction (CD28 and B7) between T lymphocytes and antigen-presenting cells, further substantiating the direct involvement of the immune response in the induction of periodontal bone resorption [72,73]. Conversely, Yamashita and colleagues transferred *A. actinomycetemcomitans*-specific Th2-cell clones to normal heterozygous rats followed by infection with *A. actinomycetemcomitans*, and found that bone loss was significantly reduced in the recipients of *A. actinomycetemcomitans*-specific Th2 cells when compared with the other infected group, which supports the notion that Th2 cells appear to interfere with periodontal bone loss [74,75]. Accumulated data also support B cell involvement in the induction of bone resorption [76]. Kozuka et al. discovered the histopathological changes in normal mice, in SCID mice that lack both B and T cells, and in B cell-reconstituted SCID mice, after repeated injections of LPS into the gingiva. As a result, the B cell-reconstituted SCID mice showed stronger inflammatory bone resorption than the SCID mice, which suggests that B cells promote inflammatory bone resorption [77]. Similarly, studies also demonstrated that adoptive transfer of antigen-specific B cells induce periodontal bone loss [78,79]. These in vivo adoptive transfer experiments of antigen-specific T cell clones [80] and antigen-specific B lymphocytes [78] firmly established the role of activated T and B cells in the periodontal bone resorption through activation and differentiation of osteoclast precursor cells along the alveolar bone surface of animals receiving antigen-specific lymphocytes.

## 4. Immune Cell RANKL Activity in the Progression and Pathogenesis of Periodontitis

Despite the complexity of the input signals, the skeletal system and bones have a relatively simple signal transduction system, capable of "sensing" a variety of stimuli, and the effector mechanism is controlled by a limited number of "key effector" pathways. In the context of alveolar bone, the system that controls bone metabolic balance includes the RANKL/RANK/OPG system secreted and recognized by specialized bone metabolism effector osteoblasts and osteoclasts [81,82].

Receptor activator of nuclear factor κB ligand (RANKL) is a cytokine that is a member of the TNF family. Its predominant function is to stimulate osteoclast differentiation, cell fusion and activation, leading to bone resorption through calcium-dependent activation of the NFATc1 gene transcription [83]. Therefore, RANKL is a major activator of osteoclasts and is a molecular signal that directly causes bone resorption. RANKL comes in two forms: (1) membrane-bound RANKL (mRANKL) and (2) soluble RANKL (sRANKL) [84]. Increasing evidence suggests that sRANKL is more potent in triggering osteoclast formation than mRANKL [85]. RANKL interacts with its receptor RANK on the surface of osteoclasts and osteoclast precursors, triggering their recruitment to the bone surface, cell fusion and activation. Many animal model experiments have shown that alveolar bone resorption can be prevented by selective inhibition of the RANKL/RANK axis [86,87]. 

Osteoprotectin (OPG) is a soluble protein that is upregulated under inflammatory conditions. As a decoy receptor, it has the function of blocking the biological activity of RANKL through competitive inhibition by limiting the ability of RANKL to bind to RANK. The quotient or ratio of RANKL and OPG determines whether conditions are suitable for bone deposition or bone resorption at any particular time. Higher RANKL/OPG ratios create conditions conducive to bone resorption, while lower RANKL/OPG ratios facilitate bone deposition [88,89]. During the bacterial-induced inflammation in experimental periodontitis, a net increase in the RANKL/OPG ratio has been shown to lead to the increased activation of osteoclasts and elevated occurrence of bone resorption [90]. There is similar evidence in the case of peri-implantitis [91], as well as pathological bone resorption of the periapical lesion due to dental pulp infection [92,93].

In periodontal disease, the inflammatory infiltration of T cells, B cells, macrophages, and neutrophils in gingival connective tissue is increased, accompanied with the increase of secretion of inflammatory mediators [94,95]. These inflammatory cells also interact with stromal cells, such as osteoblasts, periodontal ligament cells, and gingival fibroblasts. As RANKL-mediated osteoclast formation plays a key role in inflammatory bone resorption, its expression and production level were observed to increase significantly in periodontitis lesions [96]. Studies have shown that activated T and B lymphocytes are the major sources of RANKL in diseased periodontal tissues [97,98,99,100,101,102]. More than 90% of B cells recovered from human periodontal diseased tissue express RANKL, as well as about 54% of T cells [103]. These cells have also been tested in vitro to induce osteoclast differentiation and bone resorption [103,104,105]. In addition, both forms of sRANKL and mRANKL appear to be expressed from T and B cells [103]. Therefore, RANKL is now considered an important molecular signal that bridges immune response with bone metabolism.

Since the studies involving clinical tissue samples have shown that activated T and B cells produce increased RANKL in diseased sites, many researchers have sought to elucidate the mechanisms by which lymphocytes are activated and eventually contribute to RANKL-mediated bone resorption. Teng et al. used human CD4^+^ T cells isolated from local aggressive periodontitis patients and transferred them into NOD-SCID mice that have been orally colonized with *A. actinomycetemcomitans*, and found that the alveolar bone loss induced by transferred T cells was RANKL-dependent [106]. Other studies indicated that the inhibition RANKL activity by OPG reduced T cell-mediated experimental periodontal bone destruction in rodent models [103,107,108,109]. These findings further verified that antigen-specific T lymphocytes trigger bone destruction in periodontal tissues in a RANKL-dependent manner.

**Figure 2:**
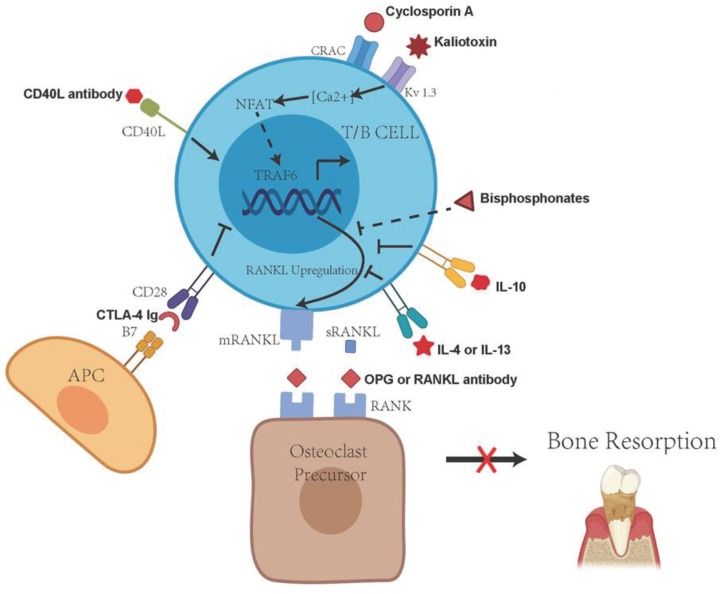
Potential immunological approaches to interfere with immune cell-mediated, RANKL-dependent periodontal bone resorption. The central target of these approaches is the inhibition of RANKL expression, secretion and interaction with RANK on osteoclast precursor cells. APC, antigen-presenting cell; CD40 L, CD40 ligand; CRAC, Ca2+ release-activated Ca2+ channels; IL-10, interleukin-10; mRANKL, membrane-bound RANKL; sRANKL, soluble RANKL; NFAT, nuclear factor of activated T cells; OPG, osteoprotegerin; RANK, receptor activator of nuclear factor κB; RANKL, receptor activator of nuclear factor κB ligand; TRAF6, tumor necrosis factor-receptor associated factor 6.

Antigen-specific T cells not only produce RANKL, but also induce B cell activation through CD40/CD40L interactions, leading to the production of RANKL by B cells [60]. In addition, there are studies showing that B cells stimulated by *A. actinomycetemcomitans* overexpress RANKL, and adoptive transfer of the antigen-specific B lymphocytes can promote periodontal bone resorption in mice injected with *A. actinomycetemcomitans* [78,79]. The observed periodontal bone resorption can be abolished by the administration of OPG-Fc in this B cell transfer model [78]. Studies of adoptive transfer of antigen-specific B cells into T cell deficient (congenital athymic) rats have shown that animals that receive antigen-specific RANKL-expressing B cells promoted the induction of osteoclasts in vitro and significantly increased experimentally induced bone resorption in vivo. Control animals receiving B cells with minimal RANKL expression did not show any bone resorption [105]. This indicates that antigen-specific RANKL-expressing B lymphocytes can promote periodontal bone resorption in the absence of T lymphocytes. Since the activated antigen-specific T and B cells are the main source of RANKL in periodontal disease, immunological interventions targeting the RANKL activities in these cells have been considered a viable approach to mitigate the immune-mediated RANKL-dependent periodontal bone loss (Figure 2).

## 5. TLR Signaling and Regulation of RANKL Activity

Regulation of RANKL production and function by TLR signaling have long been demonstrated, suggesting that interactions between TLR and RANKL/RANKL system are associated with the mechanism underlying the pathogenesis of bacteria-mediated periodontal bone loss. Zhang et al. reported that TLR2-dependent modulation of osteoclastogenesis by *P. gingivalis* is achieved through differential induction of NFATc1 and NF-κB, and the subsequent RANKL modulation [55]. *P. gingivalis* LPS could also directly induce periosteal osteoclast formation and bone resorption by stimulating RANKL in osteoblasts via TLR2. This effect may not only play an important role in periodontal bone loss but also is indicated in the enhancement of bone resorptive condition seen in rheumatoid arthritis patients with concomitant periodontal disease [110]. 

Interestingly, studies have also shown that activation of TLR (especially TLR4 and TLR9) in early osteoclast precursors leads to inhibition of RANKL-induced osteoclast differentiation through IL-12 [111]. In human osteoclast precursor cell culture models, TLR ligands inhibit RANK expression by downregulating the cell surface expression of the M-CSF receptor c-Fms, thereby inhibiting osteoclast formation [112]. As components of Gram-negative bacteria, LPS and CpG-ODN trigger TLR4 and TLR9 at the site of infection, respectively, although the biological effects of these two components may change at different stages of infection [113]. The apparent differential effects of TLR signaling on immune cells vs. bone cells in RANKL-associated osteoclastogenesis may indicate unidentified feedback mechanisms in the homeostasis of bone metabolism and such details in the interaction of the osteo-immunological network are warranted for future investigations.

Depending on the model system, outcome measurement, and microenvironment involved in such studies, different findings have also been reported regarding the engagement of TLR2 and TLR4 in *P. gingivalis* vs. ligature-induced periodontal bone loss, indicating that *P. gingivalis*-induced periodontal bone resorption is TLR4-dependent, whereas ligation-induced periodontal bone resorption is neither TLR2- nor TLR4-dependent [114]. Furthermore, the effects of TLR signaling and on RANKL-dependent and -independent mechanisms have been proposed to induce osteoclastogenesis, and other endogenous factors such as miRNA, IL-22, M1/M2 macrophages, and memory B cells have been identified recently as potential contributors in the regulation of osteoclastogenesis and bone loss during periodontal disease pathogenesis [115].

## 6. Protective Effects of Immune Regulatory T Cells in the Inflammation and Periodontal Bone Resorption

Changes in T helper cell response seem to be a key determinant of periodontal bone loss, as this type of cell not only regulates the overall immune response pattern, but may directly interfere with the RANKL/OPG ratio [116]. Regulatory T cell (Treg) is a T helper lymphocyte associated with the secretion of anti-inflammatory cytokines and resolving molecular signals such as IL-10 and TGF-β. IL-10 secretion and CTLA4 co-receptor-mediated inhibition of lymphocyte activation are hallmark effector mechanisms of Treg function, leading to active and transient suppression of the immune response and restoration of tissue homeostasis. In vivo experimental data have correlated the presence of Tregs with reduced osteolytic progression in periodontal disease [117]. In an interesting in vivo experiment, it has been shown that endogenous Tregs recruited to the specific periodontal inflammation sites can effectively reduce production of inflammatory markers in periodontal tissues, limit bone resorption, and promote the establishment of a regenerative environment [118]. It is worth noting that the selective recruitment of Tregs into periodontal tissue is not associated with an increase in bacterial load or dampening the bacterial clearance mechanism. Such Tregs modulation hence provides an attractive possibility for safe local immune regulation, which may be explored as a potential future approach to enhance the efficacy of conventional periodontal therapy [118].

## 7. Protective Effects of Immune Regulatory B Cells in the Inflammation and Periodontal Bone Resorption

Generally, B cells are considered a source of terminal effector cells that produce antigen-specific antibodies against periodontal pathogens, and they participate in the host immune response to microorganisms that invade periodontal tissue [119]. However, this view is gradually changing as we gain more insights about the role of B cells in host innate and adaptive immunity. An asynchronous relationship identified between the clinical periodontitis index and the antibody concentration in the diseased sites suggests that B cell functions may be pleiotropic in regulating periodontal inflammation [120]. Accumulated reports now suggest that in addition to T helper cells, certain subsets of B cells may be another source of immune regulators of the host immune response [121]. As early as the 1990s, existence of B cells with immunosuppressive functions were suggested. Two animal studies showed that mice lacking B cells exacerbate the disease progression in experimental autoimmune encephalomyelitis (EAE) and chronic colitis, demonstrating the potential regulatory role of B cells in autoimmune inflammation [122,123]. In 2002, Mizoguchi et al. first used the term "regulatory B cells" to describe the B cells that suppress inflammatory bowel disease [124]. B-lineage cells with regulatory functions are now commonly referred to as "regulatory B cells", or Bregs [125,126,127,128]. Currently, Bregs have been shown to have varying degrees of immunosuppressive effects in autoimmune diseases [129], allergies [130], tumors [131], transplants [132], and infections [133].

Interleukin 10 (IL-10)-producing B cells (B10 cells) are a subset of functional Bregs that inhibit experimental autoimmune encephalomyelitis, collagen-induced arthritis, and colitis inflammation [124,134,135]. B10 cells have been demonstrated to have strong potential in regulating inflammation in immune-mediated diseases and conditions in mice and humans [136,137,138]. Tedder et al. has studied the development, phenotype and effector function of mouse B10 cells, indicating that B10 cells are mainly concentrated in the spleen CD1d^hi^ CD5^+^ B cell subsets [139]. In healthy individuals, B10 cells can inhibit the differentiation of naive T cells into pro-inflammatory T helper 1 (Th1) and Th2 subpopulations, and induce their differentiation into Tregs through IL-10 secretion [140]. 

Multiple TLR signalings have been implicated in the activation of B10 cells. Recent studies have demonstrated that IL-10 production and expansion of B10 cells were regulated by TLR2/4/9 signaling in vitro [141]. We investigated the B10 activation and expansion by TLR2/4/9 agonists (*P. gingivalis* LPS and CpG ODN) using isolated spleen B cells from *P. gingivalis*-immunized and non-immunized mice and the results indicated that *P. gingivalis* LPS and CpG differentially enhance IL-10 secretion and expansion of mouse B10 cells during innate and adaptive immune responses. CpG is a strong inducer of IL-10 expression in mouse B cells, and can further induce B10 cells to secrete IL-10 [136,142,143]. Studies have found that in these responses, CD1d^hi^CD5^+^ B cells are reduced and CpG enhances IL-10 capacity more effectively than *P. gingivalis* LPS [141]. Given the dual role of TLR signaling in the activation of both pro-inflammatory and anti-inflammatory pathways in the activated B cells, it is conceivable that promoting the optimal B10 function require selective activation of TLR signaling and availability of co-stimulatory molecules.

Studies have shown that multiple co-stimulatory molecules, such as IL-21, Tim-1, and CD40 signaling are involved in the functional maturation of B10 cells [144,145,146]. These data indicated that B10 cells are activated through multiple routes of action, enhanced by antigen specificity, and can be achieved independently of TLR ligation. Yang et al. demonstrated that the combination of IL-21, anti-Tim1 and CD40L can significantly induce the IL-10 potency of B10 cells in vitro and alleviate bone loss in experimental periodontitis in vivo [147]. Similarly, Yoshizaki et al. used a mouse model and showed the differentiation and maturation of B10 cells into functional effector cells that secrete IL-10 require IL-21 and CD40-dependent homologous interactions with T cells [148]. In addition, the stimulation of CD40 and IL-21 receptor signaling can drive the development and expansion of B10 cells by a few million times and generate B10 effector cells that secrete IL-10 in vitro [136]. Furthermore, Tim-1 is essential for the induction and maintenance of IL-10 in regulatory B cells and its regulatory function on inflammation [149,150]. Xiao et al. found that Tim-1-deficient B cells had reduced levels of IL-10 production, increased levels of pro-inflammatory cytokines, and enhanced Th1/Th17 responses [150]. Tim-1 expression was detected in more than 70% of IL-10-producing B cells, and co-stimulation of B cells with alloantigen and anti-Tim-1 antibody can increase B cell Tim-1, IL-10 and IL-4 expression, potentiating the regulatory function of B cells [151]. Activation of CD40 signaling seems to induce differentiation of B10 progenitor cell population and B10 cell maturation, leading to the cytoplasmic IL-10 expression during B10 cell development, but not inducing the secretion of IL-10 [136,152]. For a long time, it has been shown that the outcome of CD40–CD40L interaction may depend on the stage of B cell maturity and the duration or intensity of reciprocal signals between T cells and B cells. These signals may ultimately determine whether these B cells can differentiate into Bregs, memory cells or plasma cells [153,154]. It has also been suggested that CD40 signaling does not induce B cell clone expansion, but rather stimulates CD1d^hi^ CD5^+^ B cells to become functionally more capable of producing IL-10 [152].

**Figure 3:**
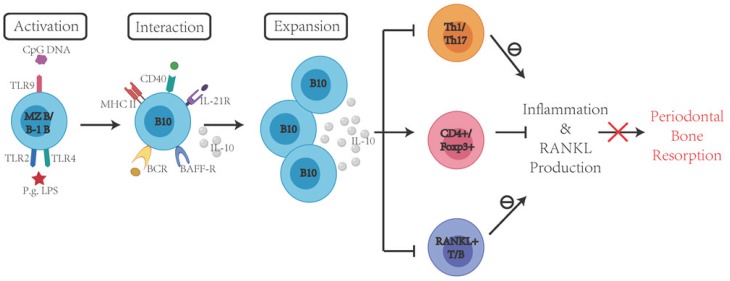
Promoting local B10 function to curtail immune-mediated periodontal inflammation and bone loss. In periodontal disease, activated Th1 and Th17 cells produce pro-inflammatory cytokines that contribute to tissue damage. Both activated T and B cells produce RANKL, which leads to osteoclast activation and alveolar bone resorption. B10 cell activation and expansion promote IL-10 secretion and promote CD4^+^FOXP3^+^Treg activation, inhibit Th1/Th17 cells and RANKL^+^ T/B cells activation, which inhibit inflammation and RANKL production, eventually alleviating periodontal bone resorption.

In vivo studies have shown that animals receiving adoptively transferred B10 cells demonstrated a reduced local inflammatory response [155], and that the local induction of IL-10 capacity of B10 cells is associated with the inhibition of inflammatory responses and periodontal bone loss in a murine model of ligature-induced experimental periodontitis in vivo [156]. Wang et al. demonstrated a significant increase in the percentage of B10 cells and the expression of IL-10 after combined treatment with *P. gingivalis* LPS (TLR2/4 agonist) and CpG (TLR9 agonist). For the first time, B10-rich CD1d^hi^ CD5^+^ B cells were adoptively transferred to mice with experimental periodontitis and the findings demonstrated that B10-rich CD1d^hi^ CD5^+^ B cells significantly inhibited RANKL production, periodontal inflammation and bone loss in a mouse model of experimental periodontitis, suggesting the regulatory role of B10 cells in the amelioration of periodontitis pathogenesis [155]. Shi et al. used purified antigen-specific B10 cells to adoptively transfer these cells into the recipient mice and demonstrated that B10 cells prevented inflammation-induced alveolar bone resorption by reducing periodontal osteoclastogenesis [157]. Moreover, compared with the control group, the mice receiving adoptive transfer of B10 cells showed reduced level of local RANKL production [157]. Evidence also indicates that adoptive transfer of B10 cells into animals can inhibit local IL-17 expression and effectively reduce the proportion of local Th17 cells [157]. It is suggested that B10 cells can regulate Treg/Th17 homeostasis during periodontitis and B10-Treg crosstalk may be involved in the regulation of local immune responses [157]. Indeed, animals with depleted B10 cells showed a significant lack of Treg cells, revealing the relationship between these two regulatory cell components [158]. Therefore, promoting B10 function locally can be a viable approach to rebalance immune response and ameliorate the progression of periodontal disease (Figure 3).

Overall, a growing number of studies have demonstrated that Bregs are crucial in the maintenance of immune tolerance and in the suppression of inflammation. Several therapeutic interventions targeting Bregs have been proposed, including ex vivo expansion of Bregs, in vivo modulation to expand Bregs, and targeted depletion of specific Breg subsets, which could provide improved approaches for the treatment of immune-mediated diseases [159]. While significant progress has been made to understand the function of B10 cells, so far most of the research focused on its role on anti-inflammatory responses through IL-10 production. However, the function of B10 is not solely mediated by IL-10 production. Moreover, although experimental evidence has clearly demonstrated the role of B10 cells in the suppression of inflammatory responses, the description of multiple Breg cell subsets and the importance of inflammatory cytokines in the induction of Breg cells raised the possibility that the Breg ontogeny is associated with the interaction between multiple B cell subsets and other cells of the immune system in response to inflammation [160]. Studies have shown that Bregs regulate CD4+ T cells and promote Tregs through both cell–cell contact and cytokines [161], and Bregs inhibit CD8+ T cell proliferation through direct cognate interactions [162]. These findings suggest that cell–cell interaction is an essential component to execute Bregs functions. 

Given the complex immune network with broad immune cell participation in chronic inflammation, the unidentified mechanism of the B10 regulatory role via cell–cell contact remains to be determined. Meanwhile, the question remains regarding the developmental link between Bregs and differentiated antibody-producing plasma cells [126]. More functional studies involving immune cell–cell interaction are needed to fully understand the mechanism of Bregs induction in vivo.

## 8. Concluding Remarks

Periodontal disease is initiated by oral dysbiosis, which lead to the immune and inflammatory responses that, on the one hand, control infection and protect the host from bacterial invasion, but on the other hand, cause collateral periodontal tissue destruction. This dual effect is reflected by the functional activities of immune cells and mediators involved in the pathogenesis of periodontitis. An increased understanding of the interactions between these cells of the immune system and the resident cells in periodontal tissue may allow us to identify new exciting targets for the treatment of periodontal disease. Among them, it appears that the activated T and B cells not only play a key role in controlling periodontal infections, but also act as a key factor in determining the extent of periodontal tissue destruction. Both human and experimental animal research support the hypothesis that by modulating the local host immune responses, there is considerable potential to interfere with periodontitis pathogenesis. Efforts are underway to evaluate strategies designed to interfere with the harmful effects of T and B cell activation to mitigate periodontal bone resorption. This may include reducing soluble RANKL activity or interfering with RANKL expression by T/B cells. Furthermore, as we gradually understand the key role of regulatory T and B cells in the control of periodontal disease progression, it will advance our understanding of the mechanisms underlying T/B cell-mediated immune regulation, and provide novel and effective targets of coordinated immune network that can be potentially modulated to achieve periodontal tissue homeostasis. Further insights into the mechanistic role of immune regulatory T and B cells in different disease settings will help us develop therapeutic strategies for better management of immune disorders.
